# How can communicative competence instruction in medical studies be improved through digitalization?

**DOI:** 10.3205/zma001350

**Published:** 2020-11-16

**Authors:** Kristina Schick, Sabine Reiser, Katharina Mosene, Laura Schacht, Laura Janssen, Eva Thomm, Andreas Dinkel, Andreas Fleischmann, Pascal O. Berberat, Johannes Bauer, Martin Gartmeier

**Affiliations:** 1 Technical University of Munich, TUM Medical Education Center, Munich, Germany; 2 University Erfurt, Professur für Bildungsforschung und Methodenlehre, Erfurt, Germany; 3 Technical University of Munich, School of Medicine, Klinikum rechts der Isar, Klinik und Poliklinik für Psychosomatische Medizin und Psychotherapie, Munich, Germany; 4 Technical University of Munich, Pro Lehre, Medien und Didaktik, Munich, Germany

**Keywords:** medical conversation, competence measurement, e-learning, digitalization, video reflection, Situational Judgement Test

## Abstract

The teaching of communicative competence plays an increasingly important role in medical education. In addition to traditional teaching formats, such as role-plays with simulated patients, technology-based approaches become more important in medical education. Teaching materials are increasingly augmented by videos of simulated doctor-patient conversations. This combination allows the content of teaching materials to be demonstrated with video or for videos to create a basis for reflection activities. In addition, conversation videos can illustrate different qualities of clinical communication and serve as illustrative material for describing particular issues in more detail. In addition to teaching clinical communicative competence, the assessment of this competence also plays an important role in medical educational research. So far, this has mainly been conducted through direct observation using checklists or rating scales. Relatively little is known about the assessment of communicative competence using standardized online-based tests. Situational Judgement Tests (SJTs) offer a promising approach in this respect. The BMBF-funded (BMBF = Bundesministerium für Bildung und Forschung – Federal Ministry of Education and Research) joint project *voLeA (Entwicklung videobasierter Lehr- und Assessmentmodule zur Gesprächskompetenz im Medizinstudium = Development of video-based teaching and assessment modules for communicative competence in medical studies)* addresses these two issues. Specifically, the project is engaged in developing e-learning modules to promote communicative competence and an assessment of this competence using an SJT. The present paper focuses on the benefits of technology-based learning and assessment units for clinical communicative competence in medical studies, using the *voLeA* project as an example.

## Zusammenfassung

In der medizinischen Ausbildung stellt die Vermittlung von Gesprächsführungskompetenz eine immer wichtiger werdende Rolle dar. Neben der klassischen Form des Rollenspiels mit Simulationspatienten und -patientinnen stehen immer öfter technologiebasierte Konzepte im Fokus der Medizindidaktik. Als didaktische Elemente dieser Konzepte werden immer häufiger Lerntexte in Verbindung mit videographierten Arzt-Patienten-Gesprächen eingesetzt. Diese Gespräche können unterschiedliche Qualitäten der ärztlichen Gesprächsführung darstellen und als Anschauungsmaterial dienen um bestimmte Sachverhalte genauer beschreiben zu können. Ebenfalls wird zur Wissensmodellierung und Reflexion angeregt. Neben der Vermittlung ärztlicher Kompetenz spielt auch dessen Assessment eine wichtige Rolle in der medizinischen Bildungsforschung. Bisher erfolgt das Assessment hauptsächlich über direkte Beobachtungen mit Checklisten oder Ratingskalen, sehr wenig ist bekannt über die Erfassung von Gesprächsführungskompetenzen mittels standardisierten online-basierten Tests. Als eine vielversprechende Methode zeigt sich der Situational Judgement Test (SJT). Basierend auf diesen beiden Punkten beschäftigt sich das BMBF-geförderte Verbundprojekt *voLeA* mit der Entwicklung einer onlinebasierten E-Learningeinheit, in denen die Gesprächsführungskompetenz Medizinstudierender durch Videomodellierung und Videoreflexion gefördert wird. Das Niveau der Gesprächsführungskompetenz wird mit einem neuentwickelten, videobasierten Situational Judgement Test erfasst. Im folgenden Beitrag soll nun das Projekt *voLeA* vorgestellt und der Nutzen von technologiebasierten Lern- und Assessmenteinheiten zur ärztlichen Gesprächsführung im Medizinstudium elaboriert werden.

## Introduction

Holding conversations with patients is one of the most common aspects of clinical practice [1]. Numerous studies have shown that the quality of communication between doctors and patients has an influence on patients' recovery as well as on the relationship with their doctors [2], [3]. Professionally conducted clinical communication also has a positive effect on doctors’ health, lowering stress levels and so reducing the risk of burnout [4]. In view of these research findings, it can be assumed that the investment in improving the communicative competence of doctors will have a direct influence on the quality of medical care [5]. 

The topic of professional clinical communication is now an integral part of the education of doctors in most human medicine curricula, but often falls short in terms of reach and scope [5]. For this reason, the German government has introduced a directive on the reform of medical studies in its 2020 Master Plan, in which it focuses on strengthening the teaching of communication with patients for (prospective) doctors [6]. The national longitudinal model curriculum Communication in Medicine [7], which comprises 350 compulsory core units and 100 elective units, serves as the framework for implementing this directive. The implementation of these extensive requirements presents most medical faculties with immense time, staffing and financial challenges. It therefore makes little sense to reinforce the communication skills of young doctors solely by expanding existing curricula. Effective teaching formats for the promotion of communicative competence offer a promising alternative since they are suitable for large groups of students and are therefore also advantageous from a resource perspective [8]. 

Developments in the area of digitalization are opening up new opportunities for devising innovative teaching approaches and making education in communicative competence for prospective doctors both more instructionally versatile and more effective. In recent years, various empirical studies have investigated and demonstrated the potential of video-based e-learning for the promotion of clinical communicative competencies [9], [10], [11], [12], [13], [14]. 

The *voLeA *(Development of video-based teaching and assessment modules for communicative competence in medical studies) joint project, which has been funded by the BMBF since 11/2018, is therefore pursuing the goal of developing digital, video-based teaching modules for the promotion of clinical communicative competence and empirically investigating their effectiveness and implementation. Within the framework of the current, first project phase, these teaching modules will be implemented in the attendance-based curriculum for clinical communicative competence at the Faculty of Medicine at the TU Munich. At the same time, a video-based Situational Judgement Test (SJT) for the assessment of clinical communicative competence will be developed in order to assess the development of communicative competence among medical students in a valid and reliable way. This paper aims to show the potential that digitalization offers for expanding and embedding communicative competence in medical studies. To this end, we summarize the current state of research on this topic and present the* voLeA* project as an example of a concrete initiative in this area. 

## Use of e-learning to promote clinical communicative competence

Competence in professional communication with patients is often referred to as communication skills in the context of medical educational research. This refers to the actual routines that (prospective) doctors should have at their disposal to ensure quality communication with patients. These include using open questions to understand a patient’s concerns or communicating relevant medical information in a clear and comprehensible way [15]. In order to build on this approach and conceptualize communication as a competence, Blömeke et al.’s definition (2015) is used in our study [16]. They define communicative competence as a bundle of different, intrapersonal resources on the basis of which communicative situations in a professional context can be successfully navigated. These resources include professional knowledge (e.g., about theoretical models of interpersonal communication), attitudes (regarding the importance of professional communication) and concrete abilities and skills (in the sense of the communication skills described above) [17]. On the basis of this definition, we can reasonably assume that a instructionally versatile course program would be useful in promoting the various aspects of communicative competence. As a recent review has shown [18], the use of digital learning programs is particularly promising in this respect. Basically, three different forms of such programs can be distinguished: 

A first group comprises video-based, interactive learning programs that use elements such as screencasts, fictional video cases and interactive exercises [10], [11], [19], [20]. These learning programs often include text-based teaching of basic communicative competence, enriched by interactive tasks or video examples. The videos also serve to illustrate successful or even faulty communication strategies and are often combined with reflective instructional items. A well-known example of this category is the doc.com platform, which is now also available in German. It comprises video-based online modules on various aspects of clinical conversations, such as delivering bad news [10]. 

Secondly, there are various programs that rely on computer-supported interaction between medical students and simulated patients (e.g., via Skype or video chat) as an instructional tool [21], [22]. Such programs involve real people assuming the role of patients and interacting with learners in real time. One such program is EQClinic, which has been shown to have a positive effect on students' ability to reflect [22], [23]. 

Thirdly, the literature discusses programs in which students interact with virtual patients [24], whom they encounter in immersive virtual reality environments such as Second Life or MPathic. These programs therefore simulate doctor-patient conversations on a virtual level. Existing studies show a high level of acceptance of such approaches among test participants [25], [26]. 

The same review [18] reports that the computer-based programs for the training of communicative competence described above have been evaluated positively and have generally achieved high acceptance ratings. In addition, all 17 studies included in the review demonstrated effects in the development of self-efficacy expectations in the communicative field as well as learning effects in the area of knowledge about communication and communicative competence. According to Muhle [18], one challenge for such programs that is evident across various studies is that virtual patients are often evaluated as unrealistic. In addition, the programs in the studies cited, without exception, represent complex Treatment Packages ([27], p. 54), which involve the interplay of different instructional elements. Consequently, most of the existing studies do not permit differentiated statements about which design elements and principles determine the effectiveness of the respective programs and how these are related to specific learner characteristics. Studies therefore make the general point that it is in principle possible to promote communicative skills with the help of e-learning. However, we need to identify and distinguish the factors influencing effectiveness more precisely. Further research in this direction is urgently needed. In the next section, the *voLeA* project will be presented, followed by a discussion of the basic instructional assumptions behind the approach pursued here. 

## Approach and objectives of the voLeA joint project

To promote the communicative competence of young doctors, the *voLeA* joint project aims to develop computer- and video-based teaching and assessment formats and analyze their effectiveness. These developments will initially be tested locally as further developments of the simulation-based curriculum at the TU Munich and (permanently) implemented there. On the one hand, this will extend an existing range of courses over time. On the other hand, there will be a diversification of the range and variety of the teaching formats used in the courses. Furthermore, the developed materials will be disseminated supra-regionally and transposed to the curricula at other faculties. 

The existing course offering is anchored in the first clinical year of study and comprises three face-to-face sessions (1.5 hrs each). These combine lecture-based elements for teaching the basics (communication models, empirical findings on the effects of good communication) with role plays in small groups with trained actors and including feedback (two actors per session). The further developed course structure (ÄGF+, Ärztliche Gesprächsführung/CCC+, Clinical Communicative Competence, duration 2.5 hrs each) is a blended learning course: Basic knowledge is imparted online in a virtual learning environment (study time approx. 45 min). The instructional phases are enriched by short illustrative videos and interactive exercises. In addition, students will watch both positive and negative examples of clinical communication, i.e., doctor-patient interactions. These form the basis for initiating reflection processes by means of guiding questions. In the subsequent attendance-based part of the course, after a short introductory sequence, the students complete role plays with feedback (as in ÄGF/CCC), whereby each small group meets three actors (duration approx. 105 min). The *voLeA* project examines the question of whether the combined ÄGF+/CCC+ course model can be used to achieve additional competence gains over the purely lecture and role-play-based ÄGF/CCC model. In order to answer this question, an SJT is currently being developed to allow a standardized measurement of communicative competence over several measurement points. In the following, we will describe the didactic design of the *voLeA* learning environment and the SJT in more detail.

## Use of videos to promote communicative competence through e-learning

The learning environment implemented in the *voLeA* project falls into the group of video-based, interactive learning programs using fictional video cases [28]. Such videos show conversational situations that are realized using scripts and actors. Compared to real videos, fictional video cases offer extended narrative and instructional possibilities: On the one hand, communicative situations can be presented realistically and serve as a basis for reflection processes [29]. On the other hand, on a narrative level, framework conditions and effects on the patients and doctors that go beyond what is shown in a conversation can be presented. For example, the history of a particular conversation or the patients' (or doctors’) reports on the conversation can be presented to relatives. Furthermore, video cases are particularly suitable for developing the ability to perceive specific situations in a professional, knowledge-based manner [30]. Two strategies that have already been used successfully in communication training are video modeling (VM) [20] and video reflection (VR) [12], [31]. The fact that the combination of these two approaches is particularly promising can be explained by referring back to the 4C/ID instruction model [32], which provides information on the effective design of complex learning environments. The authors recommend, among other things, the combination of deductive instructional procedures (as they are to be carried out within the framework of the theory-based VM modules) with inductive elements (such as reflection via video examples in the VR modules): 

### Video modeling 

Within the framework of conventional communication training with a focus on attendance-based formats, students are rarely confronted with very good examples of communicative behavior. Instead, they primarily see examples of average quality in the observation of their fellow students in role plays. However, findings indicate that video-based e-learning can be used to model very desirable as well as very detrimental communicative behavior patterns in a vivid way. By linking very good examples with targeted explanations, the development of action schemata can be promoted. In addition, video examples have a positive effect on the interest and willingness of learners to make an effort if text-based instructional stages are continuously linked with video sequences [11].

#### Video reflection

Added to this, VR enables learners to engage in an intensive mental examination of relevant examples (shown in the form of videos) [31]. This can be achieved through work assignments associated with the videos, such as identifying key points in a given conversation, justifying their selection or analyzing a scene shown based on certain criteria [12]. The comparison of one’s own communicative experiences in scenes illustrated in videos and the evaluation of video examples on the basis of basic knowledge enables learners to develop their ability to perceive communicative situations professionally [33]. There is encouraging evidence of learners reflecting on video recordings of their own simulated conversations [12] and on fictional video cases [11] in which both very good as well as problematic communicative behaviour was shown.

## Assessment of communicative competence through a Situational Judgement Test

Fundamental progress in research on communication training for medical professionals is closely linked to the development of effective assessment procedures. In educational practice for clinical conversations, competence is measured mainly via standardized simulated conversations (SG simulierte Gespräche/SCs simulated conversations, which are observed in a structured manner and evaluated using rating scales or checklists. This is done either in real-time assessments or on the basis of videographed interviews [34], [35]. Assessments of SG/SCs have established themselves as a norm in communication education in medicine and apparently have favorable psychometric characteristics [36], [http://www.aspeducators.org/]. However, they are very complicated to prepare, conduct and evaluate. This is especially true when applied to large groups of students and conducted several times by the same person to record competence development over time [37]. Furthermore, SG / SCs are complicated to evaluate. Valid and reliable evaluations require intensive training of the evaluators. Even when this intensive training is given, distortions due to the rater's differential assessment behavior and its interaction with other elements of the assessment (e.g., topics of discussion, final assessment, subjects) are difficult to rule out [37], [38], [39], [40], [41]. They can significantly affect the validity of the test scores and all other conclusions – for example, on performance feedback, developmental trajectories or group comparisons. This raises the question of how to develop more efficient assessment procedures that can complement and, in part, substitute SG/SCs. 

In particular, an SJT is being considered as a viable alternative [42]. SJTs are widespread in numerous areas of application, e.g., in staff selection. They are also particularly suitable for assessing social and non-cognitive skills, such as empathy [43]. SJT present hypothetical situations in a standardized form (e.g., in writing or on video) and require a selection or evaluation of given options for action as the response [44], [45]. For evaluation, the answers are usually compared with a solution based either on theoretical definitions or on expert empirical assessments. SJT thus record the ability of test participants to make a knowledge-based interpretation and evaluation of options for action in given situations. From a competence diagnostic perspective, such situation-specific skills are central because they mediate between individual dispositions (e.g., knowledge) and actual performance in real situations [16]. 

The *voLeA* test is to be developed as a construct-based SJT (see [46]) designed to measure clinical communicative competence and follows established procedures [44], [45]. With regard to the current context of application in the instruction of clinical communicative competence, two aspects are particularly important to us:

1. the curricular and competence-theoretical anchoring of the test concept and
2. the use of multimedia task formats for test design.

Regarding (i): First of all, the test must be valid with respect to the curriculum in order to ensure not only the validity of the content but also to promote supra-regional applicability, i.e., it must be based on standards for instruction in clinical communicative competence [7], [15], [http://www.nklm.de]. In addition, it must be linked to theoretical approaches to modeling communicative competence [41], [47]. This double anchoring is essential and valuable. Curricular standards define the concrete skills to be acquired, i.e., what performance is expected of learners at what level. The skills specified in medical curricula describe concretely observable behaviour (e.g., conversational techniques), which can serve as indicators for the development of communicative competencies [35]. However, theory-based competence models go beyond this. They describe the assumptions that underlie such manifestations of individual ability [16], [17], [48]. In addition, competence models contain (verifiable) structural assumptions about which competence dimensions are relevant and how they relate to each other. For the development of these tests, we use an approach that distinguishes three levels of communicative competence, namely

1. the ability to structure a conversation in a goal-oriented way (e.g., through techniques of metacommunication);
2. the ability to effectively advance the conversation on the content level (e.g., through techniques or strategies to create a common ground); and finally
3. the ability to build a positive working relationship with the interlocutor (e.g., through empathic behaviour) (in summary: [41]).

In other words, the model is based on the theoretical assumption that conversations are in principle to be analyzed on several levels, namely the formal-structural, the content-problem-related, and the level of interpersonal relationships [49]. To conduct conversations effectively, doctors must be able to act competently at these levels simultaneously.

Regarding (ii): Furthermore, voLeA offers the advantages of an online-based SJT format [43], [50], which allows the use of multimedia material for the construction of test items. In particular, video-based vignettes allow for a much more authentic anchoring of situations than text-only formats. This seems to be especially important in the area of communication, where not only linguistic but also numerous non-linguistic and paralinguistic cues are important for the interpretation of a situation. In fact, there is already evidence of the validity of video-based SJT for recording interpersonal competencies [42], [51]. Compared to established assessments of communicative competence, SJTs therefore offer a promising complementary perspective. The test concept developed in voLeA uses a computer-based specification of video stimuli as task stems showing excerpts from case history dialogues (approx. 45-60s length). Before watching the video, the students receive written background information on the video (e.g., name and complaints of the patients). The video then breaks off at a critical point in the conversation and the students are presented with several possible statements – related to a predefined communication goal – with which the doctor could continue the conversation. There are currently 14 tasks with corresponding video vignettes in which five alternative answers are anchored. The students’ task is to assess each of these response options in terms of their effectiveness for achieving the stated goal. The three levels of communicative competence (structure, content, relationship) are included as separate ratings. 

To ensure the validity of the content and to optimize the test format and usability, expert interviews with experienced lecturers in the field of clinical communication (n=6) and cognitive pre-tests with medical students (n=12) were conducted [52]. Subjective qualities related to test motivation (e.g., subjective learning gain, interest, effort) were also examined. The feedback obtained provides indications of a generally very good acceptance of the test procedure. For example, the experts surveyed rated the importance of testing of competency in communication skills as very high. The same applies to the approximation to real world conditions of the video situations and answer options contained in the test. The students surveyed rated the test format and its design as comprehensible and well-structured. In addition, they found the test to be interesting and appropriately challenging. On the basis of this provisional, but overall encouraging feedback, the test material was further refined. A more extensive review of the test, including psychometric quality criteria, is still pending. Various studies will be conducted by *voLeA* in the continuing course of the project.

## Outlook and planned quality assurance

The quality assurance of the e-learning modules and the SJT within the voLeA project will be conducted in the academic years 2020 and 2021. The empirical research will focus, on the one hand, on examining the effectiveness of the blended learning concept as compared to pure classroom instruction and, on the other, on the development of the communicative competence of the prospective doctors as measured by the SJT. In addition, the research will also examine which design principle of instruction via e-learning is most effective for learning – video reflection, video modeling or a combination of the two. As indicated above, studies on the psychometric quality of the SJT constitute a further research focus. In particular, the effects of various methods of test value calculation (scoring) as well as the scalability and reliability of the test values should be investigated. In addition, different aspects of validity as well as the stability of the measurement characteristics during repeated measurements will be analyzed. Besides permanent integration into the local communicative curriculum at the TUM, the teaching materials developed in the project will allow the transfer of the modules to other medical faculties. Since the SJT developed in the project is also available independently of location, this could lead to an even more differentiated research on effectiveness in the medium term: By integrating the virtual learning environment into different communicative curricula, different combinations of classroom teaching and virtual teaching units could be implemented. Thus, with the same staff resources for the exercises with simulated patients, more theoretical material for clinical communication in the form of e-learning modules could be imparted. 

Compared to assessments with SG/SCs, the SJT offers more efficient implementation and evaluation. In addition, numerous students can take the test at the same time and be assessed in a standardized way. Currently, the test evaluation is still conducted manually on a PC (e.g., using standard statistical software), but automated evaluation and feedback using appropriate algorithms is in principle possible. The test is therefore suitable as a rapid screening instrument, among other things, but also as an instructional tool for providing formative feedback to students. It should be noted that the SJT, as discussed above, primarily measures the knowledge-based perception of communicative situations and the evaluation of alternative courses of action. It thus offers a supplement to the more action-oriented assessments with SG/SCs but can by no means completely replace them. Furthermore, it is not currently intended for use for summative purposes, such as for entry selection into medical studies or for examination purposes. Although this is conceivable in principle, it does make extensive psychometric and practical demands, for example in terms of test security. 

All in all, the teaching and assessment modules developed in voLeA – either separately or in combination – can help efficiently meet the demand for more intensive training of the communicative competence of prospective doctors. 

## Funding

The joint project voLeA (development of video-based teaching and assessment modules for communicative competence in medical studies) is funded by the Federal Ministry of Education and Research from 11/2018 to 10/2021 (funding code: 16DHB2133/16DHB2134).

## Competing interests

The authors declare that they have no competing interests. 
